# On Diabetes and Its Treatment

**Published:** 1865-09

**Authors:** H. Bence Jones


### ON DIABETES AND ITS TREATMENT.

By Dr. H. BENCE JONES, A.M., F.R.S.

Each grain of starch that is taken as food, when acted on by the saliva or pancreatic fluid, is converted into sugar and becomes a source of fuel or power and ultimately passes out of the body as carbonic acid and water.

In diabetes the starch is still changed into sugar: but in the circulation and in the extravascular or parenchymatous structures the further change is partly or wholly stopped; more or less of the sugar remains as sugar, part reaches the kidneys and is thrown out in the urine, part exists in the structures unchanged; and thus the system is deprived of one part of that conversion of latent into active force on which the nutrition and power of the organ depends.

In the body or out of the body, when once a series of chemical action is set up, the series tends to become continuous; it propagates itself, unless a change of conditions occurs. For example, grape juice will remain as grape juice, if kept from the action of other substances. Under well-known conditions it will ferment, and continue fermenting, as long as sugar, and ferment, and the proper temperature are present. If the conditions are slightly varied, variations in the products will be observed. The series of actions will not pass rapidly from grape juice to alcohol, to vinegar, or to putrefaction; but according to the conditions one action or another action will be set up or will continue as long as that action is possible.

In health and in diabetes the sugar is subjected to different conditions. In health the chemical changes are propagated and continued to their full extent; whilst in diabetes the conditions necessary for perfect change are wholly or partly wanting. These conditions, on which the changes depend, are chiefly three. 1st. The proper temperature; 2d. The presence of a not immoderate quantity of sugar; 3d. The presence and activity of a ferment.

On each of these conditions a few words are necessary.

First, the internal temperature of the body, under all circumstances, is subject to so small a variation, that at present there is no proof that such a reduction can occur as can check the chemical actions in the human body, though under certain circumstances smell animals can be exposed to cold so that sugar may be made to appear in the urine.—(*Proceedings of the Royal Society, December* 15, 1864.)

Secondly, by the most careful investigation, I have satisfied myself that Professor Brücke is right in saying that healthy urine contains sugar—that is, the amount of sugar derived from the food, or from the changes going on in the textures, is at all times more than can be entirely changed in the circulation and in the tissues. If, then, a considerable amount more is thrown into the circulation, some of it will pass out in the urine. Thus an injection of sugar into the rectum when absorbed causes temporary diabetes; so, also, large quantities of sugar, fruit, and farinaceous food tend to produce diabetes. Thus, also, if Bernard's amyloid or glycogenic substance were produced in excess in the liver, diabetes would result.

Thirdly, the ferment, the prime cause of change, has not yet been insulated. Whether it be the albuminous substance of the saliva or pancreatic fluid, or some other of the many albuminous substances in the blood, it requires for its action heat, the presence of alkali, and an undisturbed circulation of the changing fluid.

With regard to the effect of alkalies, something will be said when I speak of the treatment of diabetes; but concerning the influence of unobstructed chemical action some remarkable physiological experiments must here be mentioned, because they not only furnish the most striking illustrations of the relations of mechanical to chemical disease, but because they also help to account for the effect of sudden mental shocks and nervous disturbances, which do so much harm to the diabetic patient.

Bernard made the great discovery that the mechanical injury of the floor of the fourth ventricle alters the chemical actions going on in the body so as to cause sugar in excess to appear in the urine. This is a most remarkable demonstration of the relation which subsists between mechanical and chemical actions in the living body.

M. Schiff, in his "Untersuchung uber Zuckerbildung" (1859), states that this diabetes results from injury of the vaso-motor nerves of the abdominal organs which arise from the thalami optici and the crura cerebri—*partent des couches optiques et des pedoncules cérébraux;* and he also shows that by injury of other parts of the nervous system temporary or permanent diabetes is produced.

Thus cutting the posterior roots of the nerves arising from the cervical portion of the cord, leaving the anterior roots untouched, causes temporary appearance of sugar (irritative diabetes): whilst by division of the anterior roots on a level with or above the fourth cervical vertebra, permanent (or paralytic) diabetes results. These injuries, he says, cause an irritation or a paralysis of the vaso-motor nerves. The vessels of the liver become dilated and distended; and as a consequence there is an excessive secretion of sugar by the liver. The permanent diabetes may be thus occasioned: but it is far more probable that the temporary diabetes is owing to the mechanical injury of the nervous system so affecting the vessels that the chemical changes are interrupted. The same phenomenon is seen in every fermentation. The chemical action is temporarily stopped when the fermenting fluid is disturbed by any sudden change.

These conditions of change may in diabetes be affected singly or all simultaneously. When the affection reaches to the extent of stopping the chemical change in the vegetable or animal sugar in the body, diabetes, intermittent or permanent, is the result.

That the disease is only a little way distant from health is shown by the existence of sugar in the urine in the healthy state; so that a small quantity of sugar in the urine is no proof of disease; and here, as elsewhere, there is no defined limit where health ends and disease begins.

Diabetes is the arrest of a healthy state; an increased quantity of sugar appearing in the urine, because the actions that constitute health are stopped.

In perhaps half the cases of diabetes the arrest of change in the food-sugar constitutes the complaint. This is proved by the fact that when a strict anti-farinaceous diet is observed the abnormal amount of sugar ceases, and the patient is well as long as he keeps to the strict diet. When this fact was published in 1806 by Dupuytren and Thenard, they used these words:—"Que le traitement qui consiste surtout dans un régime purement animal a le même degré d'efficacité que le quinquina dans les fièvres intermitentes."—(*Annales de Chemie*, *vol.* 59, p. 45.)

But it was soon found that cases occurred in which a strictly anti-farinaceous diet, consisting of animal food and water, did not stop the sugar in the urine. For days and weeks not a grain of vegetable starch or sugar may be taken as food, and yet sugar in excess will exist in the urine. Whence does this sugar come? It must either be taken in the animal food or it must be produced in the body.

Previous to Bernard's discoveries, this production of sugar when no vegetable food was taken admitted of no explanation. Now, the discovery of the amylaid substance in the liver, and of inosite in the muscles, lungs, brain, and other organs, gives a full solution of the difficulty. In the chemical changes going on in the body, animal starch is formed in the liver, and in other organs as in the prostate gland, and sugar in the muscles; not as in vegetables, by the fixation of carbon and the decomposition of water by the action of light, but more probably by the gradual splitting up of the higher organic compounds of the organs whilst performing their functions.

The good effect of an anti-farinaceous diet in some cases, and its want of effect in others, marks the two great divisions of the disease—the two stages in diabetes. In the first stage vegetable sugar alone ceases to go through the healthy chemical changes, whilst the animal sugar is entirely changed; in the second stage animal sugar as well as vegetable sugar are more or less unchanged. It may be concluded that animal sugar is more readily changed than vegetable sugar; and when in diabetes an anti-farinaceous diet has no marked effect, then the conditions of change of the sugar in the body are furthest from the healthy state.

There is sufficient evidence that diabetes does not always progress from one stage to the other. The disease may halt anywhere in its progress, and remain stationary for years, or get better or worse. A gentleman has consulted me for the last four years occasionally; he is now fifty-seven. When I last saw him the specific gravity of the urine was 1020, about three pints in twenty-four hours, containing about four grains of sugar to the ounce. He had been diabetic for twenty-nine years, during which time he married, and has now healthy children grown up. When he takes care about his diet, the sugar and symptoms vanish, but when careless, the symptoms return, and the sugar can be found in the urine.

Diabetes, then, in its mildest or first form, is the loss of power to change the sugar of the food; in its more advanced or more intense form, it is the loss of power to change the sugar produced in the organs and textures of the body, as well as in the food. The changes in the animal as well as vegetable sugar are arrested.

[Almost every remedy in the Pharmacopœia has been tried with the view of arresting the excretion of sugar, but none has any constant effect. Meanwhile, the effect of diet is far beyond that of any known remedy.]

An anti-farinaceous, or, in other words, an anti-saccharine diet will remove the sugar from the urine, and stop all the symptoms of the complaint in all those cases in which the power of consuming the animal sugar remains unaffected.

Even when the consumption of the animal sugar is imperfect or impossible, an anti saccharine diet will lessen the thirst, the flow of water, the dryness of the mouth, and even the constipation, and check, though it may not stop, the waste.

The simplest formula for the diet may be thus stated. All animal produce, including fish, flesh, fowl, game, eggs, cream, and meat soup should be taken; and all vegetable food that contains starch, dextrin, and sugar should be avoided.

As generally it is of the utmost importance to shun the forbidden food, I shall dwell upon it first.

The vegetable substances that contain most starch, dextrin, and sugar are rice, maize, arrowroot, sago, potatoes, oatmeal, peas, beans, bread, biscuit, toast, maccaroni, vermicelli, and all confectionery.

Fruits are even worse than vegetables. Apricots, plums, peaches, cherries, pears, gooseberries, are nearly as bad, and some worse, than rice and maize. Stout, porter, and ale, cider, port, Madeira, champagne, and sherry are more or less highly saccharine; cocoa and chocolate contain nearly 20 per cent. of starch and dextrin naturally, and more is often added.

The harm of each of these substances may be determined by the amount of starch, dextrin, and sugar they contain.

The following table, in which the fruits and farinaceous vegetables are taken as perfectly dry, will answer many questions regarding the diet of a diabetic patient:

| | Amount of Starch, Dextrin or Sugar. |
|---|---|
| Ripe dry Apricots, . . | about 93 per cent. |
| " Plums, . . . . | " 92 " |
| " Peaches, . . | " 86 " |
| " Cherries, . . | " 85 " |
| " Pears, . . . | " 84 " |
| " Figs, . . . | " 79 " |
| " Gooseberries,. . | " 37 " |
| Dry Rice, . . . . | " 90 " |
| " Maize, . . . . | " 88 " |
| " Arrowroot, . . . | " 77 " |
| " Potatoes, . . . | " 76 " |
| " Oatmeal, . . . | " 70 " |
| " Peas, . . . . | " 67 " |
| " Beans, . . . . | " 67 " |
| " Bread, . . . . | " 61 " |
| " Milk, . . . . | " 21 " |

| | | |
|---|---|---|
| Stout, . . | about 45 to 64 | grs. of sugar per ounce. |
| Porter, . | " 23 " 40 | " " |
| Ale, . . | " 12 " 45 | " " |
| Sweet Cider, | " 18 " 44 | " " |
| Port, . . | " 16 " 34 | " " |
| Madeira, . | " 6 " 66 | " " |
| Champagne, | " 6 " 28 | " " |
| Sherry, . | " 0 " 12 | " " |

If dry rice contains 90 per cent. of starch, dextrin, and sugar, and potatoes contain 76 per cent. of starch and dextrin, and if all the starch and dextrin pass off in the urine as sugar, it is evident that to forbid potatoes and to allow instead an equal quantity of rice, is simply ordering the quantity of sugar from this source in the urine to be increased 14 per cent., thus adding to the thirst and waste. Or if half-a-pint of port wine is forbidden containing from 128 to 272 grs. of sugar, and if a pint of porter or stout is ordered which contains from 368 to 960 grs. of sugar, it is clear that the quantity of sugar in the urine will thus be increased from half-an-ounce to an ounce-and-a-half daily.

Before passing to the best diabetic diet, there are two substances—bread and milk—which require to be further mentioned here.

Ordinary bread contains water, salts, starch, dextrin, sugar, and gluten.

If the salts, starch, dextrin, and sugar are washed away, the gluten remains, which, in a chemical point of view, is as unobjectionable as meat.

In making the different kinds of gluten bread this washing is more or less perfectly performed.

In the following analysis the water, starch, dextrin, and sugar were determined, the residue or difference was taken as gluten:

| | Water. | Starch and Dextrin. | Sugar. | Gluten. |
|---|---|---|---|---|
| Ordidary bread, . . | 36 | 49 | 1 | 23 per cent. |
| Aerated bread, . . | 37 | 42 | 2 | 19 " |
| Gluten bread from Toulouse, . . . | 2 | 16 to 44 | 0 | 82 to 54 " |
| Dried bread, . . . | 2 | 60 | 1 | 37 " |

The best washed gluten bread contains less starch than bran cakes or any brown bread; Dr. Pavey's almond bread is free from all starch, but the almond flour must be well washed to remove the sugar and dextrin, of which 10 per cent. are present.

Badly-washed gluten may be made into dry bread containing bulk for bulk more starch than ordinary undried bread; thus an excess of gluten bread may keep up the amount of sugar in the urine, and prevent an improvement in the symptoms.

With regard to milk, one hundred parts may be taken to contain three parts of lactine, or about half-an-ounce of lactine exists in a pint of milk. If all of this animal sugar was incapable of being consumed in the system, milk would be nearly as injurious as an equal quantity of many wines, and the best sweet ale; but experiment shows that this sugar is often partly or entirely consumed.

A diabetic patient lived upon butchers' meat alone for two two days. The quantity of urine passed was forty ounces the first, and forty-two ounces the second day; specific gravity, 1029-0. No trace of sugar could be found. He then took milk for two days; the first day eighty-eight ounces, the second day ninety-nine ounces. The urine was forty-five and a-half ounces the first day; specific gravity, 1024-1: and the second day sixty-nine ounces; specific gravity, 1011-0. Sixty-seven grains only of sugar were passed the first day, and twenty-three the second day.

At a more advanced period of the disease, when strictly animal diet did not cause the sugar to disappear from the urine, milk alone was again taken.

The first day 138 ounces of milk were drank; the urine was 61 ounces; specific gravity, 1030-5. The second day 88 ounces of milk were taken; the urine was 34½ ounces; specific gravity, 1027-8. The quantity of sugar in the urine was 854 grains the first day, and 414 grains the second day. At this time, on animal diet alone, one day 280 grains of sugar were passed, and the next day 600 grains.

Thus milk is more or less injurious according to the stage of the complaint. When animal sugar can be consumed milk is comparatively harmless.

In the advanced stages of the complaint curds or sour milk, with the acid nearly neutralized by potass, soda, or ammonia, would be as unobjectionable as gluten bread.

There are two great ends to be gained by the use of medicines in diabetes.

Of these the first and most important is to promote the oxidation of the sugar; or, failing this, to compensate the system for the loss of saccharine fuel, and the consequent loss of power and nutrition by promoting the supply and oxidation of the oleaginous fuel.

Of all the medicines that can be given for the promotion of the oxidation, whether of sugar or fat, in the body, iron and alkalies are the most energetic; and hence, beyond all other remedies, iron or ammonio-citrate of iron with excess of ammonia, or with other alkalies, are the best medicines for diabetes. This iron may be given in potass or Vichy or in Fachingen water, and that preparation which confines the bowels least is most to be preferred. Hence the potassio-tartrate and Griffith's mixture are often used.

Alkalies without iron promote oxidation. This is very evident in the copper test for sugar. M. Miahle even has stated that alkalies are the specific for diabetes; without doubt they are of importance in promoting oxidation. Soda or potass may be given in the caustic state or as carbonates. Carbonate of ammonia in ten, fifteen, or even twenty-grain doses thrice daily in any gaseous mineral lessens the thirst.

Long since Professor Graham tried the phosphate of soda, with three equivalents of soda, on the ground that the blood required this substance and could not get it in the animal food. I have not found any great advantage from its use.

Vichy water, more particularly the Celestin, is recommended by M. Bouchardat. It contains between eighty and ninety grains of carbonated alkali and alkaline earths in a quart of water. The Hospital spring contains about ninety-six grains, the Grand Gille ninety-two, the Hautrive eighty-nine, and the Celestin eighty-five grains to a quart of water.

Carlsbad Sprudel water and the more aperient Muhl spring is highly praised by Dr. J. Seegen. It contains about one-fourth or less of the alkali of Vichy water, but half a drachm or more of sulphate of soda in each quart gives an aperient action which the common salt of Vichy water rarely possesses. Marienbad Kreutzbrun water is twice as aperient, but rather less alkaline than Carlsbad Sprudel. Fachingen water has one-third or more of the alkaline power of Vichy. Seltzer water one-sixth.

Besides alkalies some animal substances are thought to promote change in the sugar in diabetes. Of these rennet and pepsine may be mentioned. In 1852 Dr. James Gray published some remarkable results in the 'Edinburgh Monthly Medical Journal,' p. 396, but I am not satisfied that rennet is very useful in diabetes. It is an albuminous substance in a state of change, and, therefore, it exactly fulfils the conditions required in the undiscovered specific for diabetes. It should be well washed with water to remove the adhering sugar and dextrin, and it should be given on an empty stomach, so as to enable it, if possible, to act on the sugar in the blood rather than on the sugar in the food.

Pepsine is another albuminous substance in a state of change. Its action in the stomach is to help the solution of the albuminous food, but when it passes into the blood it might be the animal diatase that carries on the change in the sugar. With this idea it has been given in diabetes, but with no satisfactory result; although many patients have said they were better whilst taking this remedy, yet I have never found the sugar diminish under its use. It usually helps the action of the bowels.

Lately oxygen gas has been tried by Dr. Richardson in diabetes. It would be splendidly simple if this were the specific for diabetes. This view implies that a deficiency of oxygen is the cause of diabetes, but the chemistry of the disease does not admit of this explanation. There must be a much more complex chemical error than a want of oxygen, and it is far more probable that no deficiency of oxygen exists, but that it does not act because the proper animal diastase is not present.

Vegetable and animal oils and fats constitute important remedies in diabetes. Of all these, cod-liver oil and cream are most frequently used. The following case may be taken as an instance of the amount of cod-liver oil that can be given:

A man, aged twenty-four years, was admitted into St. George's Hospital, having lost two stone in weight during eight months. He passed seven quarts of urine daily. He remained under treatment for a month, during which time he was on animal diet and cod-liver oil. He began with half an ounce daily, and this was gradually increased up to eight ounces. The quantity of urine fell to two pints and a-half, specific gravity 1030, and he increased in weight from eight st. 8 lbs. to 9 st. 1 lb.

Cream may be given in any quantity until the tongue begins to be coated, then it soon disagrees, and the stomach refuses to take it, or rejects it when taken.

Fats, combined with alkalies, as soaps, are more ready to undergo oxidation than when the glycerine is unseparated. Pure glycerine is, however, often very useful as a substitute for sugar in tea and in other liquids.

To lessen the thirst and the craving for food opium is very useful. The alkaloids of opium diffusing out of the blood in contact with the nerves of the blood-vessels cause contraction of the capillaries; this affects secretions, so that the quantity of urine, saliva, bile, and intestinal secretions is greatly diminished. The drain of urine and the desire for food are thus checked, but the increased constipation and dryness of the mouth almost, and in some instances quite, counterbalance the gain obtained by checking the flow of water. By the use of very small quantities of opium, as five or ten grains of Dover's powder, or five or ten drops of laudanum once or twice daily, the thirst and excessive flow of urine may be stopped, and the constipation may not be excessively increased.

The second great object in the treatment of diabetes is to remove the constipation.

The excessive determination of water to the kidneys causes increased dryness elsewhere, hence the mucous membrane and the skin are harsh and dry; probably also the production of acid being greatly diminished by arrest of change in the sugar, the healthy secretion by the intestines and skin cannot take place.

Notwithstanding the amount of food eaten, the action of the bowels usually is very difficult. All saline aperients increase the thirst and pass off by the urine. Magnesia from the absence of acidity is usually inactive. Castor oil is by far the best aperient, when it does not nauseate, then capsules containing castor oil with minute quantities of croton oil are most efficacious. Compound extract of colocynth with jalapine, scammony, or gamboge, or podophilline will act when oil cannot be taken. Mercury when soluble combines with the albuminous matters with which it comes in contact and sets up increased chemical action. The nutrition of the parts to which it is directly applied, or carried by absorption and diffusion, is so altered that increased action, inflammation, and ulceration are produced. This altered nutrition has no influence on the oxidation of the sugar or fat, and hence diabates is not benefited by mercury. Calomel may be used as an aperient, but it has not any advantage over other chemical or mechanical irritants to the mucous membrane of the bowels, and promoting a more rapid change in the albuminous structures of the textures causes a feebler nutrition to take place.

In extreme cases the constipation of the bowels becomes the most serious symptom. The chemical disease leads to a mechanical difficulty amounting almost to an obstruction of the bowels. Strong chemical irritants are required to excite the muscular action, sometimes even "croton oil" is necessary. When the bowels do act the prostration of the strength is sometimes alarming: so little power seems to be set free in the body that any extra expenditure seems to deprive the heart of the force necessary to carry out the circulation, in the same way as a fatiguing journey to London for advice in extreme mental anxiety will bring a prostration of strength from which with difficulty recovery takes place.—*Med. Times and Gazette*, from *Braithwaith's Retrospect.*

